# Social determinants of health, inequality and social inclusion among people with disabilities^1^

**DOI:** 10.1590/0104-1169.0187.2559

**Published:** 2015

**Authors:** Regina Celia Fiorati, Valeria Meirelles Carril Elui

**Affiliations:** 2 Professor, Faculdade de Medicina de Ribeirão Preto, Universidade de São Paulo, Ribeirão Preto, SP, Brazil

**Keywords:** Disable Persons, Social Determinants of Health, Social Inequity, Primary Health Care, Rehabilitation

## Abstract

**OBJECTIVE:**

to analyze the socio-familial and community inclusion and social participation of people with disabilities, as well as their inclusion in occupations in daily life.

**METHOD:**

qualitative study with data collected through open interviews concerning the participants' life histories and systematic observation. The sample was composed of ten individuals with acquired or congenital disabilities living in the region covered by a Family Health Center. The social conception of disability was the theoretical framework used. Data were analyzed according to an interpretative reconstructive approach based on Habermas' Theory of Communicative Action.

**RESULTS:**

the results show that the socio-familial and community inclusion of the study participants is conditioned to the social determinants of health and present high levels of social inequality expressed by difficult access to PHC and rehabilitation services, work and income, education, culture, transportation and social participation.

**CONCLUSION:**

there is a need to develop community-centered care programs in cooperation with PHC services aiming to cope with poverty and improve social inclusion.

## Introduction

In Brazil, even though some advancement has been observed in regard to the inclusion of people with disabilities in the job market and in the sociocultural sphere, there are many people who do not have access to basic rehabilitation services or equal opportunities such as access to education, vocational education, work, leisure, and other activities. Some individuals are not even aware of welfare devices and their social rights and are, therefore, restricted to the domestic environment, often in a state of social isolation^(^ 1 ^-^ 2 ^)^.

The social inclusion of individuals with disabilities is an essential requirement for health promotion and quality of life. For that, there is a need to involve these individuals and promote their participation in activities that integrate the human existential universe^(^ 3 ^-^ 4 ^)^.

The social concept of disability was the reference used in this study. This approach considers disability to be a condition that arises from the way society is constructed so that society has to reorganize itself to ensure that people with disabilities have universal access to all spaces, equipment, services, organizations and resources publicly available to the collective. Hence, services intended to promote the rehabilitation of these individuals should offer alternatives and develop strategies intended to enable their full participation in society^(^ 5 ^-^ 6 ^)^.

The acknowledgement of a biopsychosocial model of disability gains importance and relevance in 2001, when the World Health Organization (WHO) reviewed the International Classification of Impairment, Disabilities and Handicaps (ICIDH). It questions the merely biomedical approach adopted to that point, recognizing the social and political nature of disability based on the publication of the International Classification of Functioning, Disability and Health (ICF), a document that standardizes and unifies a language to describe health and its related conditions^(^ 7 ^-^ 8 ^)^.

Additionally, in 2006, the United Nations (UN) published a handbook for parliamentarians, currently in force, the Convention on the Rights of Persons with Disabilities (CRPD)^(^ 8 ^)^. The CRPD is innovative within the framework of international treaties previously agreed upon, by explicitly acknowledging that the environment, and the economic and social spheres, are factors that aggravate disabilities, while environmental and psychosocial barriers curtail the full participation of these individuals in society on an equal basis with others^(^ 9 ^)^.

In Brazil, the Federal Constitution of 1988 provides that people with disabilities are entitled to health care, public assistance, protection, social integration, and full human rights within the three spheres of government (city, state and federal); the Brazilian Health System (SUS) is also instituted as being responsible for providing healthcare to disabled individuals at the three levels of healthcare: primary, secondary and tertiary care^(^ 10 ^)^.

According to the conception that disability is a responsibility shared by society, integral healthcare provided to individuals with disabilities ranges from the prevention of diseases and the promotion of health to specialized rehabilitation, ensuring broadened and comprehensive healthcare, as provided in the National Policy of Health^(^ 11 ^-^ 12 ^)^.

There are, however, in Brazil, high levels of social inequality, submitting some segments of the population to social injustice and inequity; in this case, that is represented by a lack of access to dignified living conditions such as income, work, education, housing, and healthcare services. Studies show that people with disabilities are at a greater risk of social vulnerability when they experience difficult access to work, education and rehabilitation services^(^ 13 ^-^ 14 ^)^.

In this context, this study, as part of research conducted in Ribeirão Preto, SP, Brazil from 2011 and 2012 involving disabled individuals living in the area covered by a Family Health Center, contributes to the topic as it analyzes the inclusion of these individuals within the family (which role they played within the family) and in the community, based on their social participation and occupations. 

## Method

This study has a qualitative approach as the nature of the study object is related to the symbolic and intersubjective dimensions present in the participants, their values, the meanings they assign to their condition of health and life, cultural aspects and social representations. It is a reconstructive interpretative study because it went beyond a merely descriptive method and was based on an interpretative and reconstructive approach according to dialectical hermeneutics^(^ 15 ^-^ 16 ^)^.

After a formal introduction of the researchers, the participants and their respective families were informed about the study and signed free and informed consent forms to formalize their consent, after which, data collection was initiated. Data were collected through open interviews concerning their life histories, which were recorded with the participants' previous authorization and systematically observed by the research coordinator and students from the Occupational Therapy course administered at the Medical School, University of São Paulo at Ribeirao Preto (FMRP-USP). The individuals were asked to narrate their lives so that the biography of each interviewee was obtained. At the same time, the students took notes concerning their observations. The relationship between participants and researchers was based on respect and complied with ethical precepts. 

Ten individuals, 18 years old or older, with congenital or acquired disabilities but with cognitive conditions allowing them to narrate their life histories participated in the study. Given the study's purpose, the type of deficiency was not a criterion to select the participants, so that individuals with physical, motor, sensory, mental or multiple disabilities were interviewed because the objective was to identify the social context of disability, regardless of the clinical implications accruing from specific disabilities.

A total of 20 individuals with disabilities were identified in the area covered by the study, while the population cared for by the Family Health Service was estimated to be 250 families. Of the 20 individuals, four did not consent to participate in the study, two did not present cognitive conditions to narrate their lives, and three were not found in their homes. In the end, ten individuals met the inclusion criteria and agreed to participate.

The interviews were conducted in the houses of the individuals living in the area covered by the Family Health Center in the city of Ribeirão Preto, SP, Brazil. Prior to the interviews, the participants received clarification about the study, its objectives and methodology. The interviews lasted from one hour to one hour and 40 minutes, were recorded and transcribed afterwards. Data were analyzed and interpreted, seeking to construct knowledge based on the study objectives.

All the participants were asked to narrate their lives, from birth up to the time of the study, so that their trajectory could be reviewed. The narratives included events that were relevant to knowing their conditions and addressed their experiences contextualized with family histories. During the interviews, the following individuals were present: the participants, the researcher, family members, students from the occupational therapy course (FMRP-USP), and the researcher's advisees participating in the scientific initiation program.

The textual content from the narratives of the life histories of the interviewed individuals were interpreted in light of the Communication Action Theory of Jürgen Habermas, which is based on the assumption that communicative rationality requires a dialogical situation among the participants of a communication process, that is, there is integral reciprocity among the participants in which all linguistic utterances are equally valued. Every communicative action aims for the formation of well-founded consensus, which is defined as a mutual understanding about something in the world of life among those participating in the process. The narratives, historically and culturally contextualized in the data analysis, enabled a comprehensive and interpretative understanding of the information contained in the dialogue^(^ 17 ^)^.

Therefore, this reference enabled not only a descriptive, but interpretative and reconstructive analysis of the results. Through the ordination of data, based on reading and re-reading the life narratives, we proceeded to linking content to historical-cultural dispositions that conditioned the reports, associating data from observation in the study setting and the self-perceptions of the participants regarding their expectations concerning their autonomy, actions of healthcare services and life projects^(^ 18 ^)^.

The research project was submitted to the Institutional Review Board at the School Health Center, Medical School, University of São Paulo at Ribeirão Preto and approved on December 27, 2011 (Protocol No. 468/CEP/CSE-FMRP-USP).

## Results

The family inclusion of the individuals with disabilities participating in the study is related to the degree of social inclusion/integration that family itself occupies in society: families with better socioeconomic, cultural, and educational conditions utilize more social resources. In this sense, the individual receiving care has stronger social support and occupies a personalized place within the family; that is, the individual plays a role in the family dynamics and has an important degree of autonomy and decides on personal and family events. The social participation of these individuals is also more effective when they have access to community, cultural and social resources, which are necessary to human development. This sort of context was observed for two participants, who had completed high school and college, respectively. In the first case, the individual works selling clothes and cosmetics. In the second case, the individual receives disability retirement, having worked in qualified work with a college degree.

This more privileged condition, however, is not observed among families whose social situation tends to vulnerability, with limited socioeconomic and cultural resources, low levels of education and social support, which often lead to weakened family ties. In three of the families, social vulnerability is associated with a low level of education, lack of access to work, education, transportation, housing, and a sustainable environment. Poor hygiene conditions were found in these places in regard to both the domestic environment and the individuals, lack of information on the part of the family members and participants in regard to their disability and the care required, a lack of material resources such as assistive technology, means of transportation, experiencing financial hardships meeting basic needs, among others. 

Eight interviewees did not have significant occupations in their routines and did not have effective social participation. Four participants remained restricted to their houses due to difficult access to services, public places and a lack of opportunities that are generally available to the collective. The participants mentioned difficulties of transportation, lack of assistive technology, economic restrictions commuting to or accessing social and cultural services and devices. Even the two individuals who reported greater family support and better social conditions to leave the home, to attend public and social spaces, and to perform some domestic chores, mentioned considerable obstacles accessing public spaces due to geographic, psychosocial, and cultural barriers and also to financial difficulties accruing from being excluded from the job market. 

They also reported difficulties accessing healthcare services, both the primary and secondary levels of care, and lamented the fact that they remained without rehabilitation due to two main factors: the first involves geographic barriers and social socioeconomic and cultural conditions that lead to a lack of transportation to commute to services, lack of technical information and knowledge of social rights and lack of assistive technology to facilitate mobility; and the second factor involves the healthcare services, such as lack of healthcare delivery, lack of care performed within the community and at home, that would promote universal access for disabled individuals. There is also a lack of cooperation and connection within healthcare services, hindering coordination and the integrality of care. 

**Figure 1 -:**
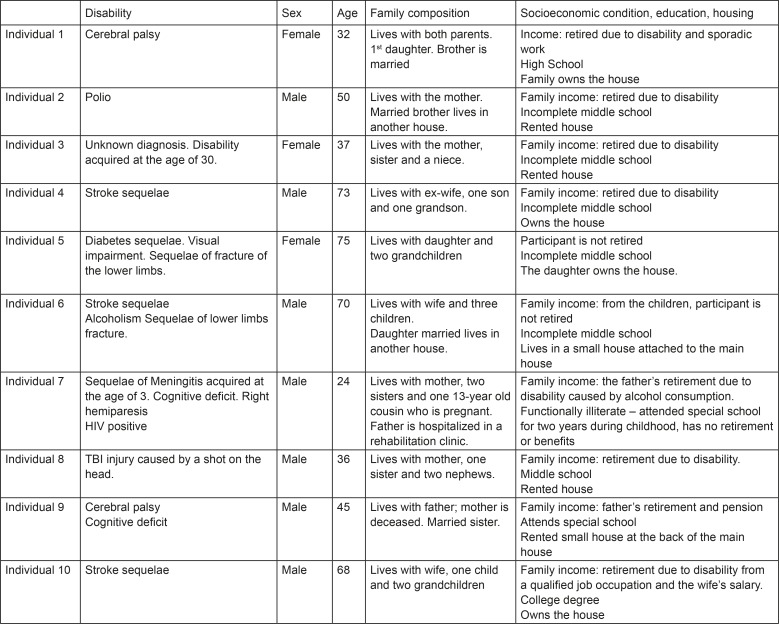
The participants' sociodemographic characteristics

Figure 1 present the participants' sociodemographic characteristics:

## Discussion

Data analysis enabled the identification of two thematic axes related to the themes predominating in the individuals' narratives: the inclusion of people with disabilities in the family and community contexts and social determinants of health and inequalities; access of people with disabilities to health services; coordination of the system from PHC to specialized rehabilitation services. 

Two thematic axes are presented and discussed below:

###  The socio-familial inclusion of individuals with disabilities and social determinants of health

The socio-familial and community inclusion of people with disabilities is directly related to economic, cultural and political factors. Social inequalities were identified in this study, evidenced by a lack of access to basic services that are essential to dignified life conditions, which leads to the discussion regarding the social determinants of health.

The situation of vulnerability is often associated with the context of people with disabilities, who are among the poorest, with the lowest levels of education and income in Brazil and in the world. The production of disabilities is directly related to poor nutrition, housing, basic sanitation, access to health services, social equipment and low income. These are determinant conditions in areas where poverty predominates, especially in poor or developing countries in Latin America, Africa and parts of Asia^(^ 19 ^)^.

Social inequality and poverty influence, and in some cases, impede access to social support networks, basic information on social rights and to resources available to the public. Additionally, people with disabilities constitute a social segment among the most excluded from the job market, from the mechanisms of income generation, education and social opportunities to equal conditions. Even though changes have been implemented in more developed nations, there are many countries with strong indexes of social inequality and unequal delivery of healthcare, directly reflecting on the life and health conditions of persons with disabilities^(^ 20 ^-^ 21 ^)^.

In Brazil, severe poverty and misery remain a concern, imposing the need to reflect upon social consequences, especially in the field dealing with families and communities, where public policies, mainly health policies and those concerning social assistance, still require more expressive actions. The State should ensure the rights of people with disabilities and provide the conditions necessary for them to have an effective role in society^(^ 2 ^)^.

This context is verified in this study and deserves greater attention in regard to its social determinants. Health technologies directed to persons with disabilities should include strategies and actions intended to interfere with these determinants, as inequalities generated in this context significantly impact the health conditions of this population. 

From this emerges the need to recover the constitutionally provided social rights of people with disabilities and the supervision of such rights so that sectors of society collaborate to develop and implement public policies intended to resolve prevalent conditions of social vulnerability in this population. It is also necessary to develop programs based on PHC to eradicate social inequalities, especially those concerning a lack of widespread access to services and opportunities. Even though are provided by Constitutional law, there is still resistance in the sphere of public administration due to income and power concentration in private sectors linked to the government and its political orientation^(^ 12 ^)^.

### Coordination of care directed to people with disabilities: from PHC to specialized rehabilitation services

According to the Brazilian Health System (SUS), healthcare delivery is organized into three levels (not considering the fourth level of care that refers to a certain type of hospital care, due to the need to more deeply address that issue than the scope of this study allows). Primary Health Care has the function to ordinate the SUS and is responsible for a group of actions that comprise health promotion, disease prevention, treatment and rehabilitation. PHC is mainly performed in the regions through community practices. The secondary level is characterized by care provided in specialized outpatient clinics, emergency rooms and university hospitals. The tertiary level refers to care delivered in large-sized hospitals^(^ 11 ^-^ 12 ^)^.

In this study, however, based on observation of the visits of community health agents and narratives of the participants, PHC services in Brazil focus on maintaining general health and few impact the disability itself, considering it to be a responsibility of specialized rehabilitation services at the secondary and tertiary levels.

Note that specialized rehabilitation services are located in places distant from the patients' residences and do not provide community-centered care or home-centered care, showing a lack of cooperation within the health care network in the region under study^(^ 1 ^)^.

The care provided to persons with disabilities is weak and lacks continuity. According to the WHO, only 2% of disabled individuals access rehabilitation services, while only 1 to 2 individuals in every 10 people access rehabilitation services in developing countries^(^ 22 ^)^.

In Brazil, people with disabilities mainly receive tertiary care, which is not in accordance with the National Policy of People with Disabilities, as that policy provides that integral care is delivered to this social segment at three levels of complexity^(^ 10 ^)^.

Another important aspect is related to the fact that rehabilitation services are exclusively guided by the biomedical and functional approach. Thus, without a social conception of disability, specialized services do not understand their responsibility concerning actions related to the social inclusion of people with disabilities, the recovery of autonomy and independence in practical and social life. This is not a problem for developed countries, which somehow manage to address most of the social determinants that affect disabilities and the accessibility of people with disabilities. Nonetheless, this is not the case for developing countries with many social inequalities and poor access to health services, which is experienced by many segments of the population, including disabled people^ (23)^.

Note, therefore, that there is a need to provide community-centered and home-centered care to these individuals. Additionally, inter-sector programs are needed to eradicate poverty and reduce social vulnerability at the local level of each region. These programs should organize and cooperate with each other and through PHC, involving local actors, cultural devices, schools, and social assistance that is available in the region, as well as government representatives and the legal sector^ (24-25)^.

## Conclusion

In Brazil, the process of the democratization of health, arising from the implementation of the SUS, included care delivered to people with disabilities in the context of public policies. Thus, after the publication of the National Policy of Health for People with Disabilities in 2002, guidelines were established for states and cities to organize their actions to provide care to these people. At this point, a broadened conception of healthcare provided to people with disabilities was recommended that included continuous care provided at three levels of complexity by cooperation among the parts of the network, promoting health, the prevention of diseases and rehabilitation, seeking to accomplish integrality in actions and beings.

Nonetheless, considering the social determinants of health and the eradication of social inequalities, there is a need to provide care in diverse local contexts, still marked by difficulties of access to material and immaterial goods and the availability of social opportunities. Thus, the creation of programs intended to provide integral care to persons with disabilities in cooperation with multidisciplinary networks and inter-sector cooperation should lead to the coordination of the various levels of care, from PHC to specialized rehabilitation services and other social devices. The Brazilian experience shows that PHC is the level that best detects the social and health needs of the population and, therefore, can mark the way for specialized rehabilitation services to proceed.

As many socially vulnerable groups are still prevalent in certain Brazilian areas, and among these groups are those with disabilities, there is a need to implement inter-sector PHC programs for vulnerable groups involving local agents from the regions identified and public service devices located in these areas, in addition to representatives of the city government to devise strategies to reduce vulnerability, create opportunities, and eradicate poverty and inequality. Therefore, once these individuals are included in these programs and strategies, they should be able to access technologies that meet their pressing needs concerning social inclusion and accessibility.

Given the previous discussion, we emphasize the importance of healthcare workers who are part of the PHC network cooperating with other social sectors to coordinate the process of developing policies and programs designed to improve the access of people with disabilities and in situations of social vulnerability to health services. Rehabilitation and opportunities to work, of generating income, finding housing, transportation, a healthy environment, quality of life and social equity are goals that should be a part of cooperation with the PHC network.
